# On Call: A Doctor’s Journey in Public Service

**DOI:** 10.3201/eid3012.241059

**Published:** 2024-12

**Authors:** Dale J. Hu

**Affiliations:** US Public Health Service, Bethesda, Maryland, USA (retired); US Centers for Disease Control and Prevention, Atlanta, Georgia, USA (1992–2013); Department of Health and Human Services, Washington, DC, USA, (2013–2015), National Institutes of Health, Bethesda, Maryland (2015–2021)

**Keywords:** Fauci, book review

For readers engaged in the prevention and treatment of infectious diseases, Dr. Anthony Fauci’s recent memoir, in which he shares not only his triumphs and achievements but also his mistakes and challenges, provides a rich resource of leadership lessons (Figure). Fauci adds depth and color to his memoir by including discussions regarding his sports interests and Italian heritage, as well as moving and deeply personal stories of his family.

**Figure:**
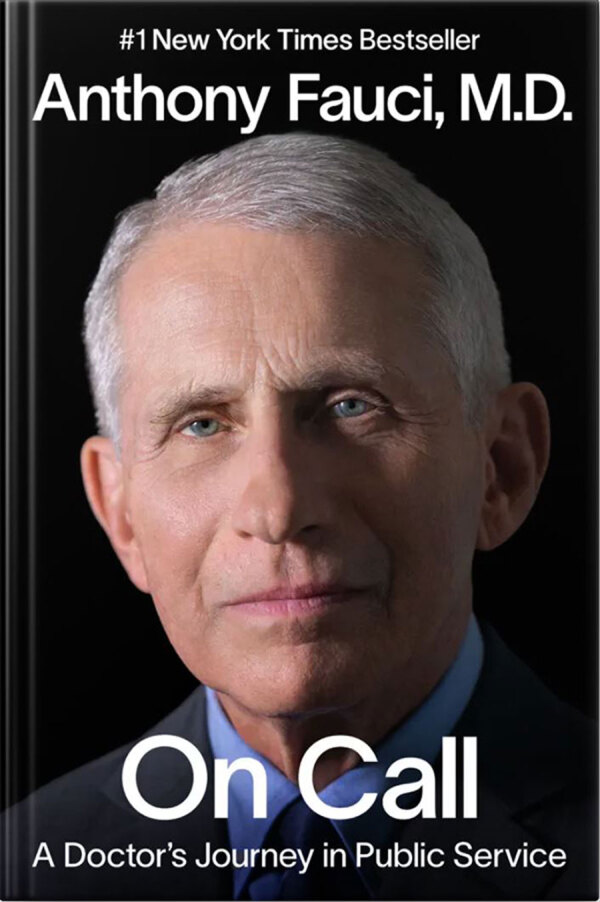
On Call: A Doctor’s Journey in Public Service

During his Jesuit schooling and subsequent medical training at Cornell University, Fauci exhibited a commitment to excellence. Starting as a focused research clinician at the National Institute of Allergy and Infectious Diseases (NIAID) at the National Institutes of Health, he soon pivoted to an enhanced leadership role in addressing the emerging HIV/AIDS epidemic, demonstrating self-confidence and superior insight. Fauci’s sharp intellect and enormous energy allowed him to successfully wear many hats: productive clinical researcher, skillful administrator, effective communicator, and inspiring leader. That multifaceted prowess has made him one of the most influential physicians and public health leaders of our time. 

As NIAID Director, Fauci steadily expanded the size and scope of the institute; the annual budget increased from $370 million in 1984 to well over $6 billion when he stepped down in early 2023 (*1*). In addition to overseeing unprecedented scientific, medical, and public health progress in addressing multiple infectious disease threats (e.g., HIV, tuberculosis, influenza, anthrax, Ebola, Zika, West Nile, and SARS-CoV-2), Fauci recruited, mentored, and supported many productive research scientists.

To support the NIAID research agenda, Fauci realized that he needed to engage and communicate effectively, noting “how important it was to cultivate relationships with people who are in a position to make things happen.” Fauci not only fostered close ties with leaders in academia, industry, and government, including 7 American presidents, but also established himself as an engaging public spokesperson.

Despite these efforts, Fauci navigated criticism, controversy, and outright hostility, especially during the HIV/AIDS and COVID-19 pandemics. With regard to HIV, Fauci effectively managed denial and criticism by engaging decision makers, activists, and the community at large to garner support for HIV research, prevention, and treatment. With the strong backing of President George W. Bush, Fauci was a principal architect for the President’s Emergency Plan for AIDS Relief (PEPFAR), on which he reflects with pride that “after more than 20 years, over $100 billion has been spent on the PEPFAR program in more than 50 countries, resulting in the saving of 25 million lives and counting.”

In the context of COVID-19, Fauci became a lightning rod for segments of society that distrusted science and the government. Although Fauci acknowledges mistakes made in the COVID-19 response, especially when information was incomplete and evolving, he emphasizes that science is an iterative process. Describing “the spread of egregious misinformation and disinformation enabled by the internet and social media” as “one of the true enemies of public health,” Fauci goes on to applaud the efforts put forth in developing highly effective vaccines, noting that “the payoff was that millions of lives were saved.” 

Fauci concludes by stating that his main motivation for writing his memoir was “to share my experiences with the world and particularly the younger generation…as an example and hopefully an inspiration for some to pursue a life serving others…” The lessons shared in this book undoubtedly provide such inspiration, along with thoughtful, intelligent strategies for combatting ongoing global infectious threats.
